# Using Item Response Theory to Identify Responders to Treatment: Examples with the Patient-Reported Outcomes Measurement Information System (PROMIS®) Physical Function Scale and Emotional Distress Composite

**DOI:** 10.1007/s11336-021-09774-1

**Published:** 2021-06-12

**Authors:** Ron D. Hays, Karen L. Spritzer, Steven P. Reise

**Affiliations:** 1 Department of Medicine, Division of general Internal Medicine and Health Services Research, UCLA, 1100 Glendon Avenue, Los Angeles, CA 90095-7394 USA; 2 Department of Medicine, Division of general Internal Medicine and Health Services Research, UCLA, Los Angeles, CA 90095-7394 USA; 3 Department of psychology, UCLA, Los Angeles, CA 90095-1563 USA

**Keywords:** individual change, PROMIS®, responders to treatment

## Abstract

The reliable change index has been used to evaluate the significance of individual change in health-related quality of life. We estimate reliable change for two measures (physical function and emotional distress) in the Patient-Reported Outcomes Measurement Information System (PROMIS®) 29-item health-related quality of life measure (PROMIS-29 v2.1). Using two waves of data collected 3 months apart in a longitudinal observational study of chronic low back pain and chronic neck pain patients receiving chiropractic care, and simulations, we compare estimates of reliable change from classical test theory fixed standard errors with item response theory standard errors from the graded response model. We find that unless true change in the PROMIS physical function and emotional distress scales is substantial, classical test theory estimates of significant individual change are much more optimistic than estimates of change based on item response theory.

**Supplementary Information:**

The online version supplementary material available at 10.1007/s11336-021-09774-1.

Randomized controlled clinical trials (RCTs) provide essential information about relative treatment effects on average. That is, a positive RCT provides evidence that at least some of the enrolled patients benefitted from the treatment (Kent et al., 2020). Two types of heterogeneity of treatment effects (HTEs) approaches have been used recently to separate patients within RCTs based on variation in benefits: (1) multi-variable modeling predicting the risk for an outcome (“risk-modeling”) and (2) evaluating interactions between treatment assignment and baseline covariates (“effect-modeling”). These approaches have been employed to evaluate clinical outcomes such as fractures, onset of diabetes, and mortality (Kent et al., 2018).

While HTE analyses are valuable in dividing the overall sample into subgroups that vary in outcomes of care, the approach still relies on group-level differences to make inferences about individuals. There is confusion in the literature about evaluating individual change (McHorney & Tarlov, 1985). For example, Coons and Cook (2018) suggested that minimally important differences (MID) based on group-level estimates be used to identify “responders” to treatment. And the U.S. Food and Drug Administration guidance document recommended identifying responders using anchor-based MID estimates, suggesting that the “difference in the PRO score for persons who rate their condition the same and better or worse can be used to define responders to treatment” (Food and Drug Administration, 2009). But standard errors for individual change are much larger than those for group-level change. Thus, using group-level indices to identify responders leads to misclassification of patients as responders when they have not actually changed. Only a few prior health outcome studies have examined individual change using the more appropriate individual-level statistics (e.g., Hays et al., 2005, 2019; Kravitz et al., 2018; Mancheño et al., 2018; McKean-Cowdin et al., 2010).

Individuals who benefit from treatment (“responders”) can be identified using classical test theory (CTT) indices such as the Jacobson and Truax (1991) reliable change index (RCI) = (${X}_{\mathrm {2}}-{X}_{\mathrm {1}})$/$\sqrt{2*\mathrm{SEM}}$, or the equivalent “smallest detectable change,” “smallest real difference,” or coefficient of repeatability (CR) $= 1.96 \sqrt{2 }^{ } \hbox {SEM} =$ 2.77*SEM, where ${X}_{\mathrm {2 }}$is the individual’s score at follow-up, ${X}_{\mathrm {1}}$ is the individual’s score at baseline, and SEM is the standard error of measurement = : $\mathrm{SD}\sqrt{1-\mathrm{reliability}}$ (Hays & Peipert, 2018). For a one-tailed test, the formula is (CR) $= 1.64 \sqrt{2 }^{ } \mathrm{SEM} =$ 2.33*SEM. The SD at baseline is typically used (Hays et al., 2005), but one can use the pooled standard errors at baseline and follow-up rather than just the baseline SEM. Stratford et al. (1996) noted that the “principal limitation of early work reporting the $\hbox {SEM}^{\mathrm {9}}$ is that this statistic assumes measurement error is constant across the range of possible scores” (p. 361). They computed conditional SEM based on the binomial theory of measurement error and a correction approach (Keats, 1962). When multi-item scales calibrated using item response theory (IRT) are used as outcome measures, IRT standard error estimates that vary by response pattern are possible (Guo & Drasgow, 2010; Jabrayilov et al., 2016; Reise & Haviland, 2005): $t={(X}_{2 }-X_{1})/\sqrt{{\mathrm{SE}}_{1}^{2}+{\mathrm{SE}}_{2}^{2}}$ , where $\hbox {SE}_{\mathrm {1}}^{\mathrm {2}}$ is the IRT estimated standard error at baseline and $\hbox {SE}_{\mathrm {2}}^{\mathrm {2}}$ is the IRT estimated standard error at follow-up. Kozlowski et al. (2016) employed a similar approach, but in the denominator, they used ($\hbox {SE}_{\mathrm {1}}+$ $\hbox {SE}_{\mathrm {2}})$/2, and this results in a smaller denominator and false rejections of the null hypothesis of no change. Lee et al. (2017) used the IRT estimated standard error at baseline only.

This paper compares estimates of change from the classical RCI that uses a fixed SEM with those based on IRT SEs. We hypothesize substantial differences between the results of these two options. Jabrayilov et al. (2016) reported that the constant CTT SEM has been shown to be too high in the tails and too low in the middle of the score distribution. But this is not necessarily always the case.

We compare the two approaches using previously collected longitudinal data with two multi-item scales in the Patient-Reported Outcomes Measurement Information System (PROMIS®) 29-item health-related quality of life measure (PROMIS-29 v2.1). The PROMIS-29 v2.1 profile assesses pain intensity using a single 0–10 numeric rating item and seven health domains (physical function, fatigue, pain interference, depression, anxiety, ability to participate in social roles and activities, and sleep disturbance) using four items per domain (Cella et al., 2019). The PROMIS-29 v2.1 profile measure is analogous to the most widely used profile measure to date, the SF-36 (White et al., 2018). But the PROMIS-29 v2.0 profile items were selected from PROMIS item banks calibrated using IRT. We evaluate the PROMIS-29 scale that best represents physical health (physical functioning) and the best measure of mental health (emotional distress) at two time points three months apart.

## Methods

We examine the PROMIS-29 4-item physical functioning scale (available online at: https://www.healthmeasures.net): (1) Are you able to do chores such as vacuuming or yard work? (2) Are you able to go up and down stairs at a normal pace? (3) Are you able to go for a walk of at least 15 minutes? (4) Are you able to run errands and shop? These items are administered without a reference period and have five response options: *Without any difficulty; With a little difficulty; With some difficulty; With much difficulty; Unable to do.* This scale is scored on a T-score metric with a mean of 50 and standard deviation of 10 in the U.S. general population (Liu et al., 2010). A higher score represents better physical functioning. The PROMIS graded response model item parameters (Table 1) for the physical functioning items were used to estimate scores. (https://www.healthmeasures.net/ is the official information and distribution site for the PROMIS measures.)

**Table 1:** Physical functioning graded response model item parameters

Item	Slope	Category thresholds			
PFA11: Are you able to do chores such as vacuuming or yard work?	4.72	$-$ 1.99	$-$ 1.53	$-$ 1.09	$-$ 0.42
PFA21: Are you able to go up and down stairs at a normal pace?	3.93	$-$ 1.90	$-$ 1.50	$-$ 1.05	$-$ 0.39
PFA23: Are you able to go for a walk of at least 15 minutes?	3.79	$-$ 1.90	$-$ 1.59	$-$ 1.20	$-$ 0.68
PFA53: Are you able to run errands and shop?	4.29	$-$ 2.62	$-$ 2.03	$-$ 1.49	$-$ 0.83

HealthMeasures is the official information and distribution center for PROMIS®.

PROMIS item parameters are available from help@healthmeasures.net.

We also present results for an 8-item emotional distress composite. Because the PROMIS-29 anxiety and depression scales intercorrelated $r = 0.82$ with one another, they were averaged together to create the emotional distress composite when the PROMIS-29 physical and mental health summary scores were created (Hays et al., 2018). The depression items are: (1) I felt worthless; (2) I felt helpless; (3) I felt depressed; (4) I felt hopeless. The anxiety items are: (1) I felt fearful; (2) I found it hard to focus on anything other than my anxiety; (3) My worries overwhelmed me; (4) I felt uneasy. These items use a past 7-day reference period with five response options: *Never; Rarely; Sometimes; Often; Always*. This scale is also scored on a T-score metric with a mean of 50 and standard deviation of 10 in the U.S. general population (Liu et al., 2010). For the analyses presented here, we employed response pattern scoring of the anxiety and depression scores using the standard PROMIS item parameters and averaged these scores together. A higher score represents more emotional distress (more anxiety and depression).

For the 8-item emotional distress composite, we used the average of the EAP SDs for the 4-item depression and 4-item anxiety scales. Table 2 shows item parameters for an 8-item emotional distress composite from a graded response model estimated for the dataset used in this study. The intraclass correlation between the average of the EAP SDs for the two scales and EAP SDs estimated from a graded response model for the eight emotional distress items was 0.92.

We use two waves of data collected 3 months apart in a longitudinal observational study of chronic low back pain and chronic neck pain patients receiving chiropractic care (Herman et al., 2018). The follow-up interval was chosen as three months based on a prior randomized trial showing small and significant improvements in SF-36 physical and mental health summary scores attributed to spinal manipulation (UK Beam Trial Team, 2004).

**Table 2:** Emotional distress graded response model item parameters

Item	Slope	Category thresholds			
EDANX01: I felt fearful	3.60	0.34	1.09	1.96	2.70
EDANX40: I found it hard to focus on anything other than my anxiety	3.88	0.49	1.26	2.11	2.90
EDANX41: my worries overwhelmed me	3.66	0.36	1.03	1.78	2.62
EDANX53: I felt uneasy	3.66	$-$ 0.23	0.60	1.56	2.50
EDDEP04: I felt worthless	4.26	0.40	0.98	1.70	2.44
EDDEP06: I felt helpless	4.14	0.35	0.92	1.68	2.47
EDDEP29: I felt depressed	4.34	$-$ 0.12	0.60	1.43	2.27
EDDEP41: I felt hopeless	4.45	0.56	1.07	1.78	2.53

Item parameters above were estimated using the dataset analyzed in this paper. The intraclass correlation between the expected a posterior standard deviations (EAP SDs) based on these parameters and the average of the EAP SDs for the depression and anxiety scales was 0.92. PROMIS item parameters are available from help@healthmeasures.net

## Analysis Plan

We categorize people into three change groups (*got worse, stayed the same, got better*) using (1) RCI based on CTT; and (2) RCI using IRT estimated SEs (expected a posterior standard deviations, EAP SDs). The CTT SEM was estimated using internal consistency reliability estimates (Cronbach, 1951). We use a pooled estimate of baseline and follow-up standard errors for both approaches so that we can isolate the impact of allowing standard error to vary across respondents.

We simulated estimated EAP scores for 10,000 observations for each measure with the following true thetas: $-3.0$, $-2.5$, $-2.0$, $-1.5$, $-1.0$, $-0.5$, 0.0, 0.5, 1.0, 1.5, 2.0, 2.5, and 3.0 using the PROMIS graded response model item parameters. This left us with 130,000 response patterns for “baseline” and another 130,000 for “follow-up.” We randomly paired each estimated baseline EAP score with a follow-up simulated EAP score to produce 130,000 simulated observations with baseline and follow-up scores.

In addition, we simulated 10,000 response patterns for change in true thetas throughout the continuum: $-3$ to $-2$, $-1$, 0, 1, 2, and 3; $-2$ to $-1$, 0, 1, 2 and 3; $-1$ to 0, 1, 2 and 3; 0 to 1, 2 and 3; 1 to 2 and 3; and 2 to 3.

Analyses were performed with SAS®, version 9.4 (2010), and simulations were conducted using R® software, version 3.5.1 (2018) and the MIRT subroutine (Chalmers, 2012). The R code is available at: https://labs.dgsom.ucla.edu/hays/pages/programs_utilities.

## Results

### Physical Functioning

**Fig. 1:**
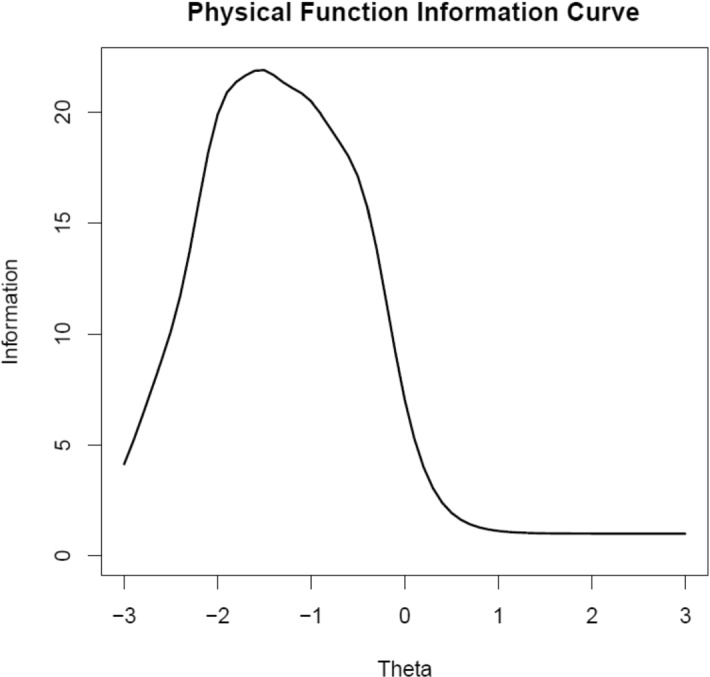
Physical functioning scale information curve

Figure 1 provides the physical function scale information curve. Information of 10 is equivalent to reliability of 0.90. This curve shows that the physical function scale has reliability of 0.90 or above for those with a physical function score in the range of average (theta = 0 on the *x*-axis) to a little below 2 standard deviations below the mean for the U.S. general population. Reliability is much lower for those with physical function better than the U.S. general population average.

**Table 3:** Percentage of individuals classified as worse, same, and better based on change from baseline to 3 months later for physical function using two-tailed and one-tailed significance tests

Reliable change index	Worse	Same	Better
Two-tailed ($p <0.05$)			
Classical test theory	173 (9%)	1425 (78%)	236 (13%)
Item response theory	56 (3%)	1677 (91%)	101 (6%)
One-tailed ($p <0.05$)			
Classical test theory	196 (11%)	1366 (74%)	272 (15%)
Item response theory	112 (6%)	1539 (84%)	183 (10%)

SEM $=$ SD * . Reliability = 0.86 ${\hbox {SEM}}_{{1}} = 2.72$; ${\hbox {SEM}}_{{2}} = 2.53$ IRT ${\hbox {SE}}_{{1}}$: mean = 3.52 (range 1.92–6.88); ${\hbox {SE}}_{{2}}$: mean = 3.61 (range 1.92–6.98)

In the same dataset, Hays et al. (2019) found significant group-level change on physical functioning $(t (df = 1833) = 4.15$, $p <0.001$), but the magnitude of change was very small (effect size = 0.08). Table 3 shows that 78% of the patients *stayed the same* according to the CTT estimates versus 91% based on IRT (two-tailed). Of the 1425 that were classified as the *same* according to CTT, 99% were also classified as the *same* by IRT (Table 4). However, only 27% of the 173 people that were *worse* according to CTT were classified as such by IRT. Similarly, only 38% of the 236 people classified as *better* by CTT were also deemed *better* by IRT. The Spearman rank—order correlation between CTT and IRT categories of change was 0.54 ($p = 0.0228$).

To illustrate why so often there was change according to the CTT fixed standard error but not by IRT standard errors, we consider one example case. There was a person whose physical functioning got worse by $-13.7$ T-score points. The RCI for the CTT was 3.7 based on the SEM of 2.6, but the RCI was 1.93 based on IRT SEs of 6.6 and 2.6 at baseline and follow-up, respectively.

According to a one-tailed test, 74% of the patients *stayed the same* according to the CTT estimates versus 84% based on IRT. Of the 1366 that were classified as the *same* according to CTT, 97% were also classified as the *same* by IRT SEs (Table 4) and 50% of the 196 people that were *worse* according to CTT were classified as such by IRT. Similarly, 58% of the 272 people classified as *better* by CTT were also deemed *better* by IRT. The Spearman rank-order correlation between CTT and IRT categories of change was 0.69 ($p = 0.0181$).

Table 5 provides mean change scores and standard deviation of change for the 7 cells with observations in Table 4. For the two-tailed change estimates, the average changes for the subgroups classified as the *same* by IRT but *worse* or *better* by CTT were substantial (− 9.72 and 9.83, respectively, on the T-score metric) but not as large as observed for those classified as *worse* ($-13.47$) or *better *(13.30) by both approaches. The average change scores for the subgroups classified as the *same* by CTT but *worse* or *better* by IRT were noteworthy but not as large (− 6.73 and 6.78, respectively).

For the one-tailed change estimates, the average changes for the subgroups classified as the *same* by IRT but *worse* or *better* by CTT were substantial (− 8.99 and 9.04, respectively, on the T-score metric) but not as large as observed for those classified as *worse* ($-11.54$) or *better *(11.63) by both approaches. The average change scores for the subgroups classified as the *same* by CTT but *worse* or *better* by IRT were noteworthy but not as large ($-5.48$ and 5.49, respectively).

**Table 4:** Cross-tabulation of change groups based on item response theory (columns) and classical test theory (rows) standard errors for physical function

Classical test theory	Item response theory			
	Worse	Same	Better	Total
Two-tailed				
Worse	**47**	126	0	173
Same	9	**1404**	12	1425
Better	0	147	**89**	236
Total	56	1677	101	1834
One-tailed				
Worse	**98**	98	0	196
Same	14	**1328**	24	1366
Better	0	113	**159**	272
Total	112	1539	183	1834

Bold indicates agreement between clasical test theory and item response theory.

### Emotional Distress

In the same dataset analyzed here, Hays et al. (2019) reported no significant group-level change on the 8-item emotional distress composite that combines anxiety and depression $(t (df = 1833) = -0.04$, $p = 0.9662$). Table 6 shows that 68% of the patients *stayed the same* according to the CTT estimates versus 90% based on IRT (two-tailed). All the 1255 that were classified as the *same* on emotional distress according to CTT were also classified as the *same* by IRT (Table 7). However, only 31% of the 290 people that were *worse* according to CTT were classified as such by IRT. Similarly, only 32% of the 289 people classified as *better* by CTT were also deemed *better* by IRT. The Spearman rank-order correlation between CTT and IRT categories of change was 0.56 ($p = 0.0172$).

Based on a one-tailed test, 64% of the patients *stayed the same* on emotional distress according to the CTT estimates versus 85% based on IRT. All the 1175 that were classified as the *same* according to CTT were also classified as the *same* by IRT (Table 7) and 44% of the 324 people that were *worse* according to CTT were classified as such by IRT. Similarly, 40% of the 335 people classified as *better* by CTT were also deemed *better* by IRT. The Spearman rank-order correlation between CTT and IRT categories of change was 0.65 ($p = 0.0148$).

Table 8 provides mean change scores and standard deviation of change for the 5 cells with observations in Table 7. For the two-tailed change estimates, the average changes for the subgroups classified as the *same* by IRT but *worse* or *better* by CTT were substantial (− 7.87 and 7.33, respectively, on the T-score metric) but not as large as observed for those classified as *worse* ($-12.34$) or *better *(12.19) by both approaches. For the one-tailed change estimates, the average changes for the subgroups classified as the *same* by IRT but *worse* or *better* by CTT were substantial (− 7.07 and 6.54, respectively, on the T-score metric) but not as large as observed for those classified as *worse* ($-11.00$) or *better *(11.11) by both approaches.

**Table 5:** Means (standard deviations) of change scores by 9 subgroups formed by cross-tabulation of item response theory (columns) and classical test theory (rows) change group in physical function

Classical test theory	Item response theory		
	Worse	Same	Better
Two-tailed			
Worse	$-$ **13.47 (3.34)**	$-$ 9.72 (1.39)	NA
Same	$-$ 6.73 (0.31)	**0.18 (2.62)**	6.78 (0.31)
Better	NA	9.83 (1.53)	**13.30 (3.94)**
One-tailed			
Worse	$-$ **11.54 (3.54)**	$-$ 8.99 (0.60)	NA
Same	$-$ 5.48 (0.24)	**0.10 (2.27)**	5.49 (0.40)
Better	NA	9.04 (0.77)	**11.63 (3.96)**

NA $=$ not applicable because there were no observations in these cells

Bold indicates for cells where classical test theory and item response theory agree.

**Table 6:** Percentage of individuals classified as worse, same, and better based on change from baseline to 3 months later for emotional distress using two-tailed and one-tailed significance tests

Reliable change index	Worse	Same	Better
Two-tailed ($p <0.05$)			
Classical test theory	290 (16%)	1255 (68%)	289 (16%)
Item response theory	90 (5%)	1651 (90%)	93 (5%)
One-Tailed ($p <0.05$)			
Classical test theory	324 (18%)	1175 (64%)	335 (18%)
Item response theory	143 (8%)	1558 (85%)	133 (7%)

SEM = SD * . Reliability = 0.93 $\hbox {SEM}_{\mathrm {1}} =$ 1.95; $\hbox {SEM}_{\mathrm {2}} =$ 1.96 IRT $\hbox {SE}_{\mathrm {1}}$: mean = 4.02 (range 2.21–6.79); $\hbox {SE}_{\mathrm {2}}$: mean = 4.01 (range 2.21–6.52)

### Classifying Change Using One- and Two-Tailed Significance Levels

Few people appear to change significantly based on IRT standard errors. Change that is not statistically significant at $p <0.05$ might still be considered worth noting if it is in the right direction. Table 9 shows an approach that might be used to reflect these concerns by incorporating both one-tailed and two-tailed tests of significance of individual change based on IRT. Those who are significantly changed based on the two-tailed test are labeled “Definitely” (Worse or Better) and those significantly changed based on a one-tailed test are labeled “Probably” (Worse or Better). Note that a very similar number of people are classified as worse versus better for emotional distress (a measure that did not change significantly at the group-level), while a greater number got *better* than got *worse* on physical function (a measure that improved significantly at the group-level).

### Simulations

Classifications of change over time based on two-tailed significance tests ($p <0.05$) for 130,000 simulated observations with random change in physical function are provided in Online Resource Table 1. When change is random, there is good agreement between CTT and IRT estimates of change. When CTT says the simulated observation got worse or got better, IRT agreed 98% of the time. When CTT classified the case as staying the same, IRT agreed 89% of the time. So, if there is no true underlying change, CTT is consistent with IRT in identifying that.

Simulated change in physical function ranging from 1 to 6 standard deviations is given in Online Resource Tables 2–15. The estimated theta distributions for true thetas of 0, 1 and 2 are similar because the information is peaked in this part of the underlying continuum (Fig. 1). The most positive response to the physical function items is most likely whenever the simulated true theta is positive.

For true theta (z-score) changes from $-3$ to $-2$, 67% of the time when CTT indicated improvement, IRT classified observations as the same (Online Resource Table 2). For larger changes from $-3$ theta (to $-1$, 0, 1, 2 or 3), CTT and IRT agreed almost perfectly (Online Resource Table 3) or exactly (Online Resource Table 4). There was good agreement about changes from $-2$ to $-1$ theta (Online Resource Table 5) and perfect agreement for changes from $-2$ to 0, 1, 2 or 3 thetas (Online Resource Table 6). Agreement was good for true changes of $-1$ to 0 theta (Online Resource Table 7), $-1$ to 1 (Online Resource Table 8), $-1$ to 2 (Online Resource Table 9), and $-1$ to 3 (Online Resource Table 10). There was almost perfect agreement for changes from 0 to 1 true theta (Online Resource Table 11) and 0 to 2 (Online Resource Table 12), and agreement was perfect for changes for 0 to 3 true thetas (Online Resource Table 13). Perfect agreement was found for changes from 1 to 2 or 3 thetas (Online Resource Tables 14–15).

**Table 7:** Cross-tabulation of change groups based on item response theory (columns) and classical test theory (rows) standard errors for emotional distress

Classical test theory	Item response theory			
	Worse	Same	Better	Total
Two-tailed				
Worse	**90**	200	0	290
Same	0	**1255**	0	1255
Better	0	196	**93**	289
Total	90	1651	93	1834
One-tailed				
Worse	**143**	181	0	324
Same	0	**1175**	0	1175
Better	0	202	**133**	335
Total	143	1558	133	1834

Bold indicates agreement between classical test theory and item response theory.

## Discussion

**Table 8:** Means (standard deviations) of change scores by 9 subgroups formed by cross-tabulation of item response theory (columns) and classical test theory (rows) change group for emotional distress

Classical test theory	Item response theory		
	Worse	Same	Better
Two-tailed			
Worse	$-$ **12.34 (3.72)**	$-$ 7.87 (1.89)	NA
Same	NA	**0.08 (2.61)**	NA
Better	NA	7.33 (1.69)	**12.19 (3.60)**
One-tailed			
Worse	$-$ **11.00 (3.70)**	$-$ 7.07 (1.77)	NA
Same	NA	**0.04 (2.37)**	NA
Better	NA	6.54 (1.53)	**11.11 (3.61)**

*NA* not applicable because there were no observations in these cells

Bold indicates for cells where classical test theory and item response theory agree.

This study shows noteworthy differences in the patients deemed to have changed versus stayed the same when using CTT versus IRT estimates of the standard error of measurement. People who changed by a substantial amount on average (12–13 T-score points for physical function and 11–12 T-score points for emotional distress) were consistently denoted as changing by both CTT and IRT. However, those who were deemed as *worse* or *better* by CTT, but the *same* by IRT declined or improved, respectively, by an average of 9–10 T-score points for physical function and 7–8 for emotional distress. The common standard error of measurement of CTT underestimates the true standard error for these individuals. Those who were classified as *worse* or *better* by IRT but the same by CTT declined or improved, respectively, by an average of 5–7 T-score points for physical function. No one was classified as changed significantly by IRT and the same by CTT for emotional distress.

The large proportion of instances in which CTT classified people as changing but IRT indicated no change indicates that which of these two approaches is used has noteworthy implications for who ends up being deemed as changed. A previous simulation study concluded that while IRT is superior to CTT in detection of individual change when a scale has 20 or more items, CTT is better for shorter scales (Jabrayilov et al., 2016). However, CTT should never be better than IRT in this respect because the raw score can never be a better estimate of true theta than the maximum likelihood or EAP theta estimate. CTT raw scores are just an approximation of the IRT model. The physical function scale examined here consisted of only 4 items, and the emotional distress composite is comprised of 8 items. If the Jabrayilov et al. (2016) study generalized it would suggest that CTT estimates might lead to better detection of true change for the PROMIS-29 scales. But the Jabrayilov et al. simulation used the Fisher information function to estimate IRT standard errors, while we used expected a posterior SDs in this study.

Some have expressed dismay at the relatively small percentage of people classified as changed based on individual statistical significance. Donaldson (2008) suggested classifying people as *almost certainly improved*, *quite likely improved*, and *probably stayed the same*. Following this idea, one could use a combination of one-tailed and two-tailed tests of significance and report five levels of change: *definitely worse* (two-tailed), *probably worse* (one-tailed), *same* (one-tailed), *probably better* (one-tailed), and *definitely better* (two-tailed). This classification preserves more information and, therefore, helps to address to some extent concerns about the lack of significant individual change.

### Limitations

**Table 9:** Number (percent) of people in different physical function and emotional distress change categories according to item response theory

	Definitely worse	Probably worse	Same	Probably better	Definitely better
Physical function	56 (3%)	56 (3%)	1539 (84%)	82 (4%)	101 (6%)
Emotional distress	90 (5%)	53 (3%)	1558 (85%)	40 (2%)	93 (5%)

Definitely worse and better groups defined as significant change according to item response theory standard errors and two-tailed test. Probably worse and better groups defined as significant change according to one-tailed test.

The single-case time-series approach for estimating individual change (Borckardt, 2008) was not entertained in this paper because most studies of health-related quality of life do not administer the survey enough times to make it practical. However, if it is feasible to do so, then that approach has the advantage of estimating variation at the individual-level rather than relying on group-level SEs.

The study is based on two PROMIS-29 measures. It is possible that results would vary with other measures. In addition, the physical function scale had ceiling effects. At baseline, 24% of the sample had the most positive possible score.

### *Conclusions and Implications*

While we analyzed data gathered from patients receiving chiropractic treatment for low back pain or neck pain, the findings are potentially applicable to other areas of research such as change in mental health associated with behavioral science interventions. This study illustrates that the amount of change in health-related quality of life scales needed to obtain statistical significance for individuals varies by location along the underlying continuum. Future efforts to identify improvement or deterioration need to use tests of significance designed for individuals and allow for measurement error to vary by where the individual is located on the underlying continuum whenever possible. If IRT estimates are not possible due to the nature of the measure or small sample sizes, then CTT estimates can be used with caution.

## Supplementary Information

Below is the link to the electronic supplementary material.

Supplementary material 1 (pdf 75 KB)

Supplementary material 2 (pdf 74 KB)

Supplementary material 3 (pdf 72 KB)

Supplementary material 4 (pdf 71 KB)

Supplementary material 5 (pdf 73 KB)

Supplementary material 6 (pdf 71 KB)

Supplementary material 7 (pdf 73 KB)

Supplementary material 8 (pdf 74 KB)

Supplementary material 9 (pdf 73 KB)

Supplementary material 10 (pdf 73 KB)

Supplementary material 11 (pdf 75 KB)

Supplementary material 12 (pdf 73 KB)

Supplementary material 13 (pdf 73 KB)

Supplementary material 14 (pdf 73 KB)

Supplementary material 15 (pdf 74 KB)
