# Supplementary material for: Using Item Response Theory to Identify Responders to Treatment: Examples with the Patient-Reported Outcomes Measurement Information System (PROMIS®) Physical Function Scale and Emotional Distress Composite
Source: Psychometrika. 2021 Jun 12;86(3):781–92. doi: 10.1007/s11336-021-09774-1 (PMC8437927; doi:10.1007/s11336-021-09774-1)
Supplement: Supplementary file 2 — Supplementary material 2 (pdf 74 KB) [file 11336_2021_9774_MOESM2_ESM.pdf]

**Online Resource Table 2. Cross-tabulation of Change Groups Based on Item Response Theory (columns) and Classical Test Theory (rows) Standard Errors for Simulated Physical Function Change From -3 to -2 Theta**

**Item Response Theory**

| **Classical Test Theory** | **Worse** | **Same** | **Better** | **Total** |
|---|---|---|---|---|
| Worse | **0 (0%)** | 38 | 0 | 38 |
| Same | 0 | **445 (100%)** | 0 | 445 |
| Better | 0 | 6,417 | **3,100 (33%)** | 9,517 |
| Total | 0 | 6,900 | 3,100 | 10,000 |

From: Using Item Response Theory to Identify Responders to Treatment: Examples with the Patient Reported Outcomes Measurement Information System (PROMIS®) Physical Functioning and Emotional Distress Scales
*Psychometrika*
Ron D. Hays, Karen L. Spritzer, Steven P. Reise; University of California, Los Angeles
Corresponding Author: Ron D. Hays: drhays@ucla.edu
