# Supplementary material for: Using Item Response Theory to Identify Responders to Treatment: Examples with the Patient-Reported Outcomes Measurement Information System (PROMIS®) Physical Function Scale and Emotional Distress Composite
Source: Psychometrika. 2021 Jun 12;86(3):781–92. doi: 10.1007/s11336-021-09774-1 (PMC8437927; doi:10.1007/s11336-021-09774-1)
Supplement: Supplementary file 5 — Supplementary material 5 (pdf 73 KB) [file 11336_2021_9774_MOESM5_ESM.pdf]

Online Resource Table 5. Cross-tabulation of Change Groups Based on Item Response Theory (columns) and Classical Test Theory (rows) Standard Errors for Simulated Physical Function Change From -2 to -1 Theta

Item Response Theory

**Classical Test Theory**	**Worse**	**Same**	**Better**	**Total**
Worse	**0 (0%)**	2	0	2
Same	0	**99 (100%)**	0	99
Better	0	1,189	**8,710 (88%)**	9,899
Total	0	1,290	8,710	10,000

From: Using Item Response Theory to Identify Responders to Treatment: Examples with the Patient Reported Outcomes Measurement Information System (PROMIS®) Physical Functioning and Emotional Distress Scales
Psychometrika
Ron D. Hays, Karen L. Spritzer, Steven P. Reise; University of California, Los Angeles
Corresponding Author: Ron D. Hays: drhays@ucla.edu
